# Hospital and Institutional News

**Published:** 1915-01-02

**Authors:** 


### HOSPITAL AND INSTITUTIONAL NEWS.

### THE VOLUNTARY HOSPITALS BRIGADE.

The inquiries which still arrive at this office concerning the Voluntary Hospitals Brigade show not merely how numerous are the women, and men, too, for that matter, who have been waiting for an organisation of this kind to give them an opportunity of helping at the present crisis, but also the desirability of again reminding our readers that the aims and objects of the Brigade were fully described in The Hospital of November 28, pp. 185-6, and December 5, p. 215. As soon as the arrangements which we are now completing are in working order, those intending members who have already communicated with us will be informed. But we again repeat there is no need for them in the meantime to remain idle. All those who have recognised the need for assisting the hospitals in the great work which is being done by them at the present time, under which their resources are being severely strained, should make an active attempt to secure as many recruits for the Brigade as possible. These should be asked to become members of the Voluntary Hospitals Brigade, and to undertake to raise £1 a head per annum so long as the war lasts, or else to contribute to the League of Mercy, when called upon, such small sums of money as 1s. and upwards. We repeat these directions as a convenient summary of the purpose which the Brigade should serve. All the money raised will be given to the hospitals through the League of Mercy, and so will be directly of value to the sick and wounded. All experience has shown the value of this snowball system, and the welcome already accorded to the Brigade proves that it provides a means for these women who are genuinely anxious to do necessary work to become, as they wish, of practical assistance to their country at the present time.

### BOMBARDMENT OF THE HARTLEPOOLS.

The escape from destruction of the Hartlepools Hospital during the recent bombardment is very remarkable. The hospital stands quite exposed to the sea and immediately behind the batteries. Though shells fell all around, none actually struck the building. At the time of the engagement the hospital was filled with patients, who were all in a few minutes removed from the wards to that part of the ground floor on the safest side of the building. Hardly was this accomplished than the wounded began to pour in—women, children, men, terribly shattered, dead, and dying. Numbers were treated in the out-patient department and many admitted to the wards. Eighteen of these died within a few hours. The whole of the honorary staff, with the welcome assistance of Professor R. Morison, of Newcastle, responded heartily to the call, and were fully employed until late in the evening. Two tables were in constant use in the operating theatre, and in all over forty serious operations were performed, including many amputations. Moreover, the matron and entire nursing staff rendered valuable services during a most trying day, for they themselves had undergone the terrible anxiety of possible destruction during the bombardment. One must also bear in mind the most opportune assistance of the many voluntary helpers, men and women, trained and untrained.

### SECRETARY GUARDING GERMAN PRISONERS.

Among the provincial hospital secretaries now serving in His Majesty's Forces is Mr. G. A. H. C. Borgnis, secretary of the Newbury District Hospital, who has received a commission as second-lieutenant in the National Reserve. Latest reports state that he is stationed on H.M.S. *Lake Manitoba*, which is one of the vessels employed off the South Coast for confining suspected aliens and German prisoners. As a result of this exchange from administering a hospital for the sick to guarding a centre for prisoners on the part of Lieutenant Borgnis, an emergency secretary has been appointed—namely, Sir Frederick Montagu Pollock, who is undertaking the duties temporarily, and possibly, if he remains in the district, during the entire period of absence of the secretary. At the time when the appointment was made Mr. W. Holding, the chairman, pointed out that it had been necessary to summon an emergency meeting of subscribers because "there was no provision in existence for a secretary who by force of circumstances was not in a position to perform his duties." Sir Frederick Montagu Pollock, who is the third holder of the baronetcy, is fifty years of age, and was described by Dr. Heywood as being specially qualified for the post. The hospital possesses over thirty beds and cots, and in an ordinary way is administered by two honorary secretaries in addition to the officer now absent on duty.

### METROPOLITAN HOSPITAL SUNDAY FUND.

The forty-second report of this Fund for the year 1914 records a marked improvement in its economic administration. Sir Ernest Tritton, Bart., has proved himself an able chairman of the Finance Committee. Under his vice-chairmanship the Fund has progressed, for, despite the difficulties of 1914, the total collections in the churches have exceeded those of the previous year. There was a reduction in the working expenses of about £500, the total of which fell from a sum equal to 4⅞ per cent. of the gross receipts to 3.9 per cent. This is a remarkable reduction in the circumstances. We believe that these encouraging economies and the marked improvements in the administration will greatly increase the popularity of the Hospital Sunday Fund in London. In these circumstances the collections in the churches and chapels should speedily revert to, or even exceed, the highest standard of previous years—viz. £50,000 per annum. The great amount of work which the London hospitals are doing for the nation by maintaining numerous beds for the sick and wounded, who are being received in increasing numbers, will give the voluntary hospitals in 1915 a paramount claim upon the gifts of all classes throughout the metropolis of the Empire. We report in this issue the proceedings at the annual meeting of the constituents of this Fund, which was largely attended and passed off with unanimity and success.

### THE HISTORIAN OF THE ROYAL ARMY MEDICAL CORPS.

We regret to announce the death, at the age of seventy-one, of Colonel William Johnston, C.B., M.D., of the Army Medical Service (retired.) An Aberdeen man, educated at Aberdeen and Edinburgh Universities, he entered the Army Medical Service in 1865, and after service in the Zulu and Transvaal campaigns of 1865 and 1881, he retired in 1892. The South African war, however, saw him re-employed as Assistant Director of the Army Medical Service, for which he later received promotion and the C.B. Always interested in historical research and compilations, Aberdeen University owes to him its "Roll of Graduates from 1860-1900," and at the time of his death he was busily engaged on a very laborious and detailed biographical history of the Royal Army Medical Corps. Always at work to improve and confirm its status in the Army, which we cannot doubt will be fully recognised in even greater measure by the present war, he was seen at work on this crown of his endeavours at the Public Record Office in London only a few days before his death. The importance of histories of this character we have done our best to emphasise in The Hospital for many years, and we can only hope that so careful a compiler has placed the material at his disposal and his ripe knowledge in the hands of a competent literary executor, so that his service may be completed, and the Royal Army Medical Corps may receive, but, alas! posthumously, the history which it richly deserves, and for which Colonel Johnston worked so steadily.

### SHOULD MEDICAL OFFICERS DISPENSE?

We have previously drawn attention to the fact that it is desirable in all large hospitals and infirmaries that a properly qualified dispenser should be appointed to dispense medicines. We are informed that at the new Halifax Poor-Law Hospital—which institution we reported on in our issue of December 12—the resident medical officer advertised for is required to dispense all medicines for some 400 patients, a small extra salary being given for this work. In our suggestions given for improvements in the staff required and remuneration (The Hospital, December 12, page 244) we said that "a hospital of 400 beds should be placed in charge of a resident medical officer who should have an assistant resident medical officer—at present there is only an assistant medical officer, and the payment made to that officer, £140 per annum is at least £110 too small." We would now further suggest to the Halifax Guardians that they should appoint a qualified pharmacist as dispenser, who would be wholly responsible for the manufacture and dispensing of medicines.

### SCIENTIFIC PHARMACY IN LARGE INSTITUTIONS.

By this means, provided the salary offered for the resident medical officer (advertised for by the Halifax Guardians as vacant) is increased to a more adequate amount, it should be less difficult to obtain applications from suitable candidates for this responsible post. Scientific pharmacy, as practised at our large hospitals, is something infinitely more than the "mixing together of things in a bottle." An experienced dispenser can be of great assistance to the medical staff, by preparing sterile solutions for injection, staining solutions, etc., and making many galenicals and special preparations, many of which, it is admitted, are more reliable when made in the hospital dispensary than when bought by tender at very low prices.

### AMBULANCE BARGES FOR FLANDERS.

We have already drawn attention to the part played for ambulance and hospital purposes by barges on the rivers of France, and now understand that Sir Arthur Sloggett, K.C.B., Surgeon-General of the British Forces, has accepted the proposal of Mr. Douglas Hall, M.P., to procure and equip four ambulance barges for immediate use on the waterways in Flanders and France. The donor is understood recently to have experimented on the Seine with the use of barges both for hospital and transport purposes, to the satisfaction of the authorities. The new unit of four barges, which will be forthcoming if the public response to the plan and generous gifts are adequate to defray the expense, will be at first under the control of the Royal Army Medical Corps, which will provide the staff, and, it is understood, a floating hospital complete for 200 wounded. The small committee which has been formed to carry out the plan has as its chairman Mr. S. A. Grant, M.P., and cheques, payable to the "British Water Ambulance Fund," should be sent to him at 29 St. James's Street. Mr. Douglas Hall is going out to the Front to confer with Sir Arthur Sloggett on matters of detail.

### A POOR-LAW CHRISTMAS CARD.

Acute observers may often find among the cartloads of Christmas cards each year a few of genuine originality and merit. We ourselves drew attention to a remarkable example of hospital interest twelve months ago; but it has been left to an original mind to devise this year a Christmas card which has found its inspiration in, of all things, the Poor Law. The card consists of a four-page leaflet in an art-paper cover, bearing the words "Christmas Hymn, 1914." Within, on page 1, are two quotations from the *Times* and the *Star* respectively. The former of these records the famous decision of the Lambeth Board of Guardians to dock the children of their institution of an egg on Christmas morning (since, we believe, rescinded); the latter that of the same Board of Guardians to continue unabated the customary annual increases in the salaries of certain officers in their employment. Then follows

J. C. Squire's satirical poem on the egg episode, reprinted from the *New Statesman*. This indirect incitement to those feelings of humanity, generosity, and thought for others, merged together in personal service, for which the festival of Christmas stands, is very happily expressed in this original production. We draw attention to it, however, not only because of its institutional origin, but also as reminding institutional workers themselves of the possibilities (not necessarily of a satirical kind) which their own hospital or infirmary offers for original Christmas cards on future occasions. There can be no doubt that much pleasure might be given to friends, and much interest be aroused in the institution, if everyone interested in hospital work would take the hint contained in the private card which we have mentioned, and make his or her hospital the central idea of Christmas cards next year. A good personal reason for doing this is also that in the mass of Christmas cards sent out now annually, only a genuine piece of originality can really give a personal stamp and personal pleasure to those who give and receive them.

### "KING ALBERT'S BOOK."

Always a good friend to our voluntary hospitals, whose developments and needs it records faithfully and well, the *Daily Telegraph* may be sincerely congratulated on having secured the pen of Mr. Hall Caine to describe last Tuesday his visits to hospitals on Christmas Day. Apart from the facts of a hospital Christmas, and the fine atmosphere of the wards on this festival, Mr. Hall Caine uses all his rhetorical power to describe the happiness caused to the soldiers by their Christmas presents, including, perhaps most remarkably, the gift of "King Albert's Book" from the *Daily Telegraph* and Sir Francis Trippel. No one who read his fervent article but will echo his wish that the pleasure given by this book may be extended to every wounded soldier at home or in the base hospitals; and this can be done if the public cooperate in the movement. The pictures, both coloured and otherwise, are a stirring record, and will remain a tribute not merely to the present crisis, but to the thoughtfulness of a great newspaper, and the Press in general, for our voluntary hospitals to-day.

### THE MECHANISM OF MODERN WAR.

All recent talk of Armageddon and the greatest war of the world overlooks the fact that mere size fails to appeal to the human imagination, and that Waterloo or any small but famous battle of history (La Rothière is a good example) is far more moving than any incident so far in the war, except possibly the retreat from Mons. We are bored by the millions of men, the monstrous calibre of the heavy guns, and would exchange them all for a fight between two monoplanes manœuvring for the upper position. Indeed, the wearisome mechanism of modern warfare is well brought out in a recent letter from "An Officer in the R.A.M.C.," published in the lay Press. After describing the trenches, in which, he well says, "one feels like Gulliver walking along a Lilliputian town all the time," he breaks off by saying, "I was just starting for my daily constitutional on top when the enemy began their bombarding, one and a half hours earlier than usual." What a light this throws on the mechanical nature of modern war! Bombardment, it almost comes to, from ten to four, interval for tea, aeroplane attack, and so on. If our victory, which we believe is sure, puts an end to war, it will be at a time when science has carried its engines of destruction and the methods resulting from them to a pitch of absurdity. Think for a moment that at the very time when heavy guns are believed to have made all fortifications virtually useless, siege warfare in the trenches is the main feature of modern battles. Efficiency carried to this pitch stultifies itself, and the fact is interesting to hospital men as a reminder that there is and must be a limit to the size of their own institutions, and to the number of beds contained in them, if efficiency is not in hospital work also to cut its own throat.

### DEATH OF SIR ROBERT M. SIMON, M.D, F.R.C.P.

Sir Robert Michael Simon, for twenty-three years honorary physician at the Birmingham General Hospital, died at his residence in Edgbaston last week after a brief illness. In the medical life of Birmingham Sir Robert occupied a prominent position, being identified with Hammerwich Cottage Hospital, Tewkesbury Hospital, Nuneaton Hospital, Sutton Coldfield Dispensary, and the Birmingham Dental Hospital. He graduated at Caius College, Cambridge, taking his B.A. degree. After studying medicine at Guy's and at Berlin, he took his M.B. in 1875, and twelve years later his M.D. He obtained the Fellowship of the Royal College of Physicians (London) in 1895. In the academic life of the city the doctor held the posts of Professor of Medical Jurisprudence at Mason College, and when the Faculty of Medicine was taken over by the University of Birmingham, Sir Robert became Professor of Therapeutics. He was a frequent contributor to medical literature. Sir Robert married a daughter of the late Mr. W. H. Willans, and that lady is a first cousin of the Prime Minister, whose host the doctor had invariably been when Mr. Asquith visited Birmingham. Sir Robert received his knighthood in June 1910, and only in April this year resigned office as senior physician, and on October 14 last was honoured by the governors and subscribers of the hospital (as reported in The Hospital on October 24), the occasion being the presentation of an illuminated address in recognition of thirty-five years' continuous service. At the same time a handsome diamond tiara was given to Lady Simon, and a portrait of the doctor was presented to the hospital to be hung in the board room.

### THE ST. JOHN AMBULANCE BRIGADE HOSPITAL.

The Army Council has approved an offer from the Order of the Hospital of St. John of Jerusalem in England to provide and maintain for service, whether abroad or at home, for not less than six months, a general, fully equipped hospital of 525 beds, to be called the St. John Ambulance Brigade Hospital. The military authorities are to find a suitable building, quarters, and staff, and the Chief Commissioner of the Brigade will be in command. The entire *personnel*, with the exception of a few special appointments such as trained hospital nurses, cooks, and dispensers, will be selected from duly qualified members of both ambulance and nursing divisions of the Brigade. Each district in the Brigade will be asked to send a proportionate number of volunteers. The probable cost of each bed will be £100 for a year, and the name of each donor will be displayed on a tablet at its head. Any collective body contributing sufficient to maintain twenty-five beds for a year—namely £2,500—is to be entitled to have a ward named after it. All members of the Order and Associates are asked to use every effort in their power (not only by personal contribution but by endeavouring to obtain donations from every available source) to make it a complete success. The proposal has the concurrence of the Joint Committee of the Order of St. John and of the British Red Cross Society, and the Chief Commissioner at the seat of war, Surgeon-General Sir Arthur Sloggett, K.C.B., a Knight of Grace of the Order, has promised that when the time arrives a suitable site shall be found for it, preferably abroad. A sub-committee has been appointed to assist in promoting the new institution, and consists of the Chief Commissioner of the Brigade, Col. Sir James Clark, Bart., F.R.C.S., surgeon-in-chief, Mr. Edmund Owen, and the lady superintendent in chief of the nursing corps and divisions, Lady Perrott.

### TANNERIES AND INFECTIOUS DISEASES.

The question as to whether tanneries act as antidotes during epidemics of infectious diseases has been raised at Bermondsey, the home of the leather industry in London. During the serious outbreaks of scarlet fever and diphtheria in the Metropolis in the last few months there has been a remarkable immunity from such diseases in Bermondsey. This is borne out by figures. In seven weeks the medical officer stated that 73 notifications of scarlet fever were received and 35 of diphtheria, whilst in the neighbouring borough of Southwark the figures were scarlet fever 243 and diphtheria 45; Lambeth, 298 scarlet fever and 88 diphtheria; Greenwich, 113 scarlet fever and 49 diphtheria; Camberwell, 288 scarlet fever and 62 diphtheria; and Lewisham, 191 scarlet fever and 52 diphtheria. The meagre figures, as compared with other London boroughs, are due, according to the opinion of certain medical experts, to the tanneries, which are considered to act as antidotes to outbreaks of infectious diseases. Whatever may be the truth with regard to this hypothesis, it is nevertheless a fact that not only is Bermondsey particularly free from infectious diseases at the present juncture, but that during the cholera epidemic of some few years ago in London the borough was one of those which practically escaped.

### THE HOSPITALS AND WAR RISKS.

One of the unforeseen effects of war, which even prophets of Armageddon might have overlooked a few months ago, is the risks of war to hospital buildings. Instances—and since the Scarborough and Hartlepools raid we hardly need reminding—are to hand of the way in which institutions, particularly on the East Coast, insured themselves against this danger soon after war began; and no doubt, since the raid, coast town hospitals generally have been making up their minds on this matter. The Great Yarmouth Hospital, whose situation appeals to the imagination as being, we may say, in the centre of the danger zone, has insured itself to the extent of £40, and no doubt the date of its action has given it better terms than would be now obtainable. It would be interesting to know the extent to which insurance has been undertaken, and we should be glad to receive particulars upon this head. Meanwhile, where this has been reasonably carried out, the fact that it has been deemed advisable to insure might usefully be brought before the public as an easily appreciated instance of the exceptional needs of the voluntary hospitals at the present time.

### HONOUR FOR A HOSPITAL SECRETARY.

Two noteworthy facts concerning the officers and administration of the Worcester General Infirmary were dealt with on December 21. The post of medical officer was decided to be thrown open to women doctors, as the Chairman explained that the honorary staff did not object, and that the only man who had applied for the post did not accept it, on the ground that the number of beds was not sufficient. The Dean further explained that during the war it was impossible to get men at the remuneration offered by the infirmary. The second matter arose when the ordinary business was over. The Bishop of Worcester then presented Colonel Stallard, the former secretary to the infirmary, with an illuminated address, which carried with it the distinction of a life governorship of the infirmary. During Colonel Stallard's term of office, as the Bishop remarked, in 1887 the new wing had been added and opened. In another decade the Nurses' Home had been built, and in little more than another decade the annexe in memory of King Edward VII. had been added. The era had not only been strenuous but anxious, because during those years the financial difficulties of the infirmary had come about. Colonel Stallard had been faithful in his trust, punctual in his business, and courteous to them all. They were giving Colonel Stallard something which did not bring him in any advantage at all. It was a thing which brought advantage to the infirmary. Colonel Stallard, in the course of his reply, remarked that the invested funds of the infirmary were now almost exactly what they were when he took up his duties as secretary thirty years ago. He thought the infirmary had now started a fresh career of usefulness, which would be manifest and highly appreciated in the future.

### WOMEN DOCTORS AND INFIRMARY POSTS.

In some quarters the old prejudice against women doctors is long dying, and among those who share it must apparently be included the Hammersmith Guardians, one of whose last acts during the past month was to reject a proposal to appoint temporarily a fully qualified medical woman to a post at Hammersmith Infirmary at a salary of four guineas a week. This suggestion, those interested in social questions may like to note, was made by Mrs. Cobden Sanderson, but the chairman, Mr. C. Pascall, replied that it would be an innovation which should first be carefully considered by a committee. Colonel Davies remarked that the Guardians would be wiser to appoint the woman doctor than an unqualified young man. Eventually the whole subject was referred to a committee, while the difficulty of obtaining a fully qualified man remained unsolved. If the committee makes inquiries they will find, if they do not know the fact already, that qualified medical women have held and given satisfaction in a variety of Poor-Law institutional posts. The arguments against them doing so were discussed in The Hospital of September 7, 1912, by a medical woman in one of these positions, and she recorded her experience that the difficulties which critics had led her to expect had solved themselves practically without much trouble.

### A SCARCITY OF MEDICAL WOMEN.

A remarkable outline of the difficulty experienced in late years, and more particularly during the past twelve months, in filling resident medical posts, was given recently by Mr. W. H. Harper, house governor and secretary of the Wolverhampton General Hospital, to a meeting of the Hospital Saturday Committee. He remarked that six members of the honorary staff of doctors had been lost owing to the war, and in addition another had been compelled to relinquish his duties because his partner had gone. He could well remember the time when it was quite a common thing when they advertised for a house surgeon or physician to get from ten to twenty applications for one post. Things gradually got worse, however, until, advertise as they would, they absolutely failed to get any applications for the post vacant. In May he had hurriedly to call a special meeting because in a few days' time they were to have been without a resident medical man, notwithstanding five or six weeks' advertising. He (the speaker) was sent off to London and visited nine of the leading medical schools, and the result was that after extreme difficulty the resident post was filled. As soon as ever it was known that medical officers were wanted in the Army and Navy all the resident medical staff volunteered. On two occasions it had been impossible for members of the honorary staff to see all the out-patients. He had gone to London again, and did the round of the medical schools. He could not find a single man who wanted employment as a doctor, and theirs was not the only hospital in such a position. Well, they had to have somebody in residence, and they had had to fall back on medical ladies. From inquiries he had made none of the hospitals who had lady resident doctors had ever had reason to regret it. It might be thought that there were plenty of medical ladies in England, but a tour of the principal trading centres showed that there was a great scarcity. The outcome of his search was that three lady resident doctors had been engaged. One of them was already in residence, and two others arrived on January 1. Their resident staff now consists of one man and three ladies.

### TEACHING HOSPITALS AND PHARMACOLOGICAL CLASSES.

The new edition of the British Pharmacopœia comes into force this week, and hospitals will have to consider the question of revising their own special Pharmacopœias in accordance with the new standards and the altered strengths of many frequently prescribed medicaments. In the great teaching hospitals the classes for pharmacology are generally held in the summer session, and the students taking the course this year will naturally require to be taught according to the new official standards. In the dispensaries of these hospitals it is probable that the 1898 Pharmacopœia will serve for some months yet—in fact until the special and individual hospital Pharmacopœia has been revised and corrected, mixtures prescribed under the names used in those Pharmacopœias will presumably, *ipso facto*, be made up with B.P. 1898 tinctures, etc. In actual practice the necessary alterations are not so very great in number, but several of them are of very great importance—for example, the tinctures of nux vomica and strophanthus. Considerable confusion would be obviated and the interests of the coming generation of practitioners would be advanced if these alterations were made immediately at those hospitals which have medical schools attached to them.

### THE NEW YEAR'S REFERENCE BOOKS.

The time has again come for us to scrutinise the up-to-dateness of the reference books which now form a permanent feature of every secretarial department, and the degree of recourse to which is one criterion of the alertness and enterprise of each administrative office. Thus each year which passes gives a new, indeed a double, interest to Burdett's "Hospitals and Charities," which becomes to those who study its chapters as well as refer to its tables and directory, the most convenient critical summary available of all the events which form the features of each number of The Hospital. Probably for everyday use and clerical purposes Black's "Who's Who," when read in the light of its complementary volume, the "Who's Who Year Book," takes the premier place. Indeed, it is interesting to remark the healthy tendency which is ceasing to regard such directories of names as "Who's Who" as mere channels for ambitious advertising, since hospital secretaries and men of affairs generally have come to count on obtaining ready information on all public points in any man's career. Every profession really should have its register, and the appearance of any name in its appropriate volume will one day be regarded as natural as is its appearance among the list of telephone subscribers. In this sense, as at once the cause and the effect of civilised conveniences, medical and other directories have played a useful educational part. The "Medical Who's Who," published by the London and Counties Press Association, Ltd., price 10s. 6d. net, now that it has wisely become purely inclusive, continues to grow in size, and the alphabetical order in which the names appear has made it, apart from professional purposes, the readiest reckoner of its class. The "Englishwoman's Year Book and Directory," price half a crown, points to the enlarged sphere of social and institutional work now open to women, and Black's "Writers' and Artists' Year Book," in days when hospital managers have also to possess journalistic gifts and experience, is extremely useful. "Whitaker's Almanac" is the bible of the man of affairs, but, like other bibles, it is, in common with other works of reference, one much bought but little read. Almoners, perhaps, particularly among institutional officers, will find Herbert Fry's "Royal Guide to the London Charities" (price 1s. 6d.) of much value, for the alphabetical particulars of the very numerous organisations mentioned in it give at one glance the chief points which an institutional reader requires to know. The only defect a brief examination shows is the reproduction in the preface of the current nonsense about Nietzsche, which is quite inexcusable as being entirely irrelevant to the subject. Since it is only ignoramuses who forget that the main feature of his work was a hatred of Prussia and her militarism, we regret that a useful publication should have so unnecessarily committed itself.

### THE SALE OF LAUDANUM.

One curious effect of the new Pharmacopœia, which came into force on January 1, 1915, will be to make it more difficult for the public to purchase laudanum. Hitherto this preparation has been classified among poisons in the second part of the Poisons Schedule, and provided the chemist who sold it placed a poison label and a name and address label on the bottle, his legal obligations were fulfilled. The new laudanum, however, contains 33 per cent. more opium than the old, and this is just sufficient to bring the preparation into the first part of the Poisons Schedule, one of the items in which is "opium, and all preparations or admixtures containing 1 or more per cent. of morphine." Under these circumstances, laudanum may only be sold to persons known or introduced to the seller, and an entry of every transaction must be made in the poisons book and signed by the purchaser of the laudanum. No doubt these extra precautions will serve to impress upon the customer the potent nature of the substance, and if they have the tendency to diminish the sale of laudanum, a salutary purpose will be served. There can be no doubt that in some districts the practice of laudanum drinking has not completely disappeared, and while the new conditions of sale can hardly be expected to put an end to the remnants of this noxious habit, they may be hoped to do something in the proper direction. The purchase of pennyworths of laudanum for use in home-made cough mixtures will also be discouraged by these precautions.

### DRUG HABITS AND THE NEW PHARMACOPŒIA.

The idiosyncrasies of individuals to drugs and the effect of the publication of the new Pharmacopœia were well illustrated in the inquests heard by Dr. Waldo in the Southwark and City Coroners' Courts last week. In one case an institutional cook was stated to have taken four or five ounces of laudanum every week for five or six years, supplied to her by one of Boots' drug stores, the manager of which said that he had continued to serve her because she did not seem to be affected by it. The witness also pointed out that the law only required the poison to be supplied in a bottle distinguishable by touch and duly labelled. Dr. Waldo recalled the case of De Quincey, with his several thousand drops a day, remarked that the manager had carried out the law's requirements, and stated that he felt sure that the jury would agree with him in congratulating the General Medical Council on the new edition of the British Pharmacopœia, in which, from January 1, laudanum was placed in Part I. of the Schedule of the Pharmacy or Poisons Acts. Part I. requires that the purchaser must sign the poisons book, while the drug itself will be stronger. In another case an Indian woman (who had lived with a man who stated that he did not believe in marriage, but believed mutual trust and confidence between men and women was sufficient, for eighteen years), was found to have died by misadventure. She had, it appeared, like nearly all Indians, taken drugs, like opium or cocaine, ever since she was a child. This habit, which she had given up at times, was revived of late, and the man she lived with returned one day to find her unconscious in their room at a London hotel. Death, which occurred on the way to hospital, was due, according to medical evidence, to heart failure while suffering from cocaine and morphine poisoning. The Coroner advised a police inspector to secure the names and addresses of the chemists who had sold bottles unlabelled.

### THIS WEEK'S DRUG MARKET.

Business has been practically at a standstill since the last report appeared, and will not be resumed in earnest until stock-taking is over. The advancing tendency in the price of cod-liver oil continues, and so far as can be foreseen there is not likely to be any movement in a downward direction for the present. The future of the market depends mainly on the results of the Norwegian cod fishings which will shortly commence, and some anxiety is felt with regard to the effect of the war on the fishings; an increased demand from Germany for cod-liver oil, and an increase in transport charges from Norway to this country, are two of the factors which account for the recent rise in price. Among the articles which will be watched with interest when the market commences to be active again are opium, morphine, codeine, mercurials, carbolic acid, salicylates, citric acid, tartaric acid, and quinine.

